# Diabetes Mellitus Is Associated with a Lower Risk of Gout: A Meta-Analysis of Observational Studies

**DOI:** 10.1155/2020/5470739

**Published:** 2020-07-09

**Authors:** Xiaoli Li, Lianju Li, Yuling Xing, Tiantian Cheng, Shaohui Ren, Huijuan Ma

**Affiliations:** ^1^Department of Rheumatology, Xingtai People's Hospital Affiliated to Hebei Medical University, Xingtai 054001, China; ^2^Department of Internal Medicine, Hebei Medical University, Shijiazhuang 050017, China; ^3^Department of Endocrinology, Hebei General Hospital, Shijiazhuang 050051, China; ^4^Department of Medicine, Xingtai People's Hospital Affiliated to Hebei Medical University, Xingtai 054001, China; ^5^Hebei Key Laboratory of Metabolic Diseases, Hebei General Hospital, Shijiazhuang 050051, China

## Abstract

**Aims:**

Although several epidemiological studies have investigated the relationship between diabetes mellitus (DM) and the risk of gout, the results are inconsistent. Therefore, we systematically retrospected available observational studies to clarify the impact of DM on the risk of gout.

**Methods:**

Embase, PubMed, Cochrane Library, Scopus, Web of Science, and China National Knowledge Infrastructure were searched for relevant articles from inception to 2 March 2020. The quality of the included studies was assessed using the Newcastle-Ottawa Quality Assessment Scale. The multivariate adjusted relative risks (aRR) and corresponding 95% confidence intervals (CI) were pooled based on a random-effect model. Cochran's *Q* test and *I*^2^ were used to evaluate heterogeneity.

**Results:**

Five studies involving 863,755 participants were included in our meta-analysis. DM was associated with a lower risk of gout (aRR: 0.66; 95% CI: 0.59 to 0.73) but had a high heterogeneity (*I*^2^ = 89.2%). Metaregression analysis revealed that the types of DM were the source of heterogeneity. Subgroup analysis by types of DM showed that the risk of gout was significantly lower in type 1 DM (T1DM) (aRR: 0.42; 95% CI: 0.28 to 0.63) than in type 2 DM (T2DM) (aRR: 0.72; 95% CI: 0.70 to 0.74). Furthermore, when stratified according to gender in DM, sex-specific association was found. The inverse association was observed in males only (aRR: 0.57; 95% CI: 0.43 to 0.77) and not in females (aRR: 0.96; 95% CI: 0.87 to 1.05). Further stratified based on glycated hemoglobin (HbA1c) levels in DM, raised A1C levels were associated with a reduced risk of gout in patients with DM.

**Conclusions:**

This meta-analysis indicated that DM was related to a lower risk of gout, and the protective effect of DM on the risk of gout was stronger in males, T1DM, or DM with high HbA1c levels. However, more prospective cohort studies are required to confirm these results.

## 1. Introduction

Gout is a crystal-associated arthropathy characterized by the deposition of monosodium urate (MSU), which is directly related to hyperuricemia caused by disorders of purine metabolism and/or decreased uric acid excretion. The prevalence of gout accounts for approximately 5% of the middle-aged and elderly global population, and the incidence of gout has increased steadily in recent years [1, 2]. Individuals with T2DM generally have a higher prevalence of high blood pressure [3], obesity, and decreased kidney function [4]. These comorbid conditions are also risk factors of gout. Both DM and gout are related to a high risk of cardiovascular events, kidney failure, and mortality [4–7]. Therefore, the relationship between DM and gout has attracted great attention.

Several prospective studies found that gout was positively associated with the risk of DM [8–11]. Similarly, a meta-analysis of 11 cohort studies with 42,834 participants reported a positive correlation between serum uric acid level and the risk of DM [12]. However, the impact of DM on the risk of gout was inconsistent. Several small cross-sectional studies showed that DM was associated with a higher risk of gout [13–16], whereas a prospective cohort study [11] and a case-control study [17] suggested that DM was negatively correlated with the risk of gout, and no association was found in another prospective cohort study [18]. Therefore, we retrospected available observational studies to clarify the impact of DM on the risk of gout.

## 2. Methods

### 2.1. Search Strategy

This meta-analysis was registered in PROSPERO with the registration number CRD42020159645. Six databases including Embase, PubMed, Cochrane Library, Scopus, Web of Science, and China National Knowledge Infrastructure (CNKI) were searched for relevant articles by two authors (X-L L and Y-L X) independently from inception to 2 March 2020. The search strategy was the combination of the MeSH terms and entry terms for “Gout or Gout arthritis” and “DM or T1DM or T2DM.” Meanwhile, the searched studies were limited to human beings and there was no restriction in languages. Taking PubMed and Embase databases for example, the details of the retrieval process are listed in supplementary material Excel S1-S2.

### 2.2. Eligibility Criteria

The purpose of this study was to explore the impact of DM on the risk of gout. Our eligibility criteria are as follows: (1) Studies should have an observational design and should investigate the relationship between DM and the risk of gout. (2) Studies should diagnose DM prior to the diagnosis of gout. (3) Outcomes should be presented as the multivariate adjusted relative risk (RR), odds ratio (OR), or hazard ratio (HR) with corresponding 95% CI. (4) Studies should involve subjects without DM or gout as the corresponding control group.

### 2.3. Study Selection and Data Extraction

The screening of articles was independently performed by three authors (X-L L, L-J L, and T-T C). For inconsistent results, we would discuss them together or solicit the final judgment of the senior researcher (H-J M). Standardized data sheets were used by the abovementioned investigators to independently collect data. The following information was extracted: first author, year of publication, study design, data source, study period, definition of cases and controls, ascertainment of DM/gout, follow-up period, numbers of cases and controls, sex ratio, ages of cases and controls, types of DM, adjustment confounders, and adjusted OR/RR/HR (95% CI). All entries were confirmed by two of the authors mentioned above and checked at least twice to ensure accuracy and completeness.

### 2.4. Study Quality

The quality of the included studies was assessed using the Newcastle-Ottawa Quality Assessment Scale (NOS) [19]. As a quality assessment tool, NOS evaluated the quality of a study through three aspects: 4 stars for selection, 2 stars for comparability, and 3 stars for exposure/results, with a total of 9 stars for case-control studies and cohort studies. A score of 0-5 was regarded as low quality, while a score that reached six or more stars was considered to be high quality [20]. To ensure accuracy, the process of quality assessment was conducted by two authors independently and supervised by the senior researcher (H-J M).

### 2.5. Statistical Analysis

As the incidence of gout was relatively low (<5%) [1], the OR of a case-control study or the HR of a cohort study was used as an estimate for RR to compute the pooled RRs [21]. The multivariate adjusted relative risks (aRR) and corresponding 95% CI reported in the studies were used to produce forest plots in our meta-analysis. Heterogeneity was evaluated by Cochran's *Q* test and *I*^2^. The degree of heterogeneity was judged as follows: 0% < *I*^2^ ≤ 25% represented insignificant heterogeneity, 25% < *I*^2^ ≤ 50% indicated low heterogeneity, 50% < *I*^2^ ≤ 75% showed moderate heterogeneity, and *I*^2^ > 75% represented high heterogeneity [22]. If *I*^2^ < 50%, the heterogeneity between groups was low and a fixed-effect model was used. Whereas, for *I*^2^ > 50%, the heterogeneity was obvious and a random-effect model was used. Metaregression analysis and subgroup analysis were performed to explore the source of heterogeneity. To identify sources of heterogeneity and assess the robustness of the results, sensitivity analysis was conducted by removing each study individually and calculating a pooled effect estimate for the remaining studies to assess whether a single study affected the results. All statistical analyses were conducted with STATA 14.0 software.

## 3. Results

### 3.1. Literature Search Results

**Figure 1:**
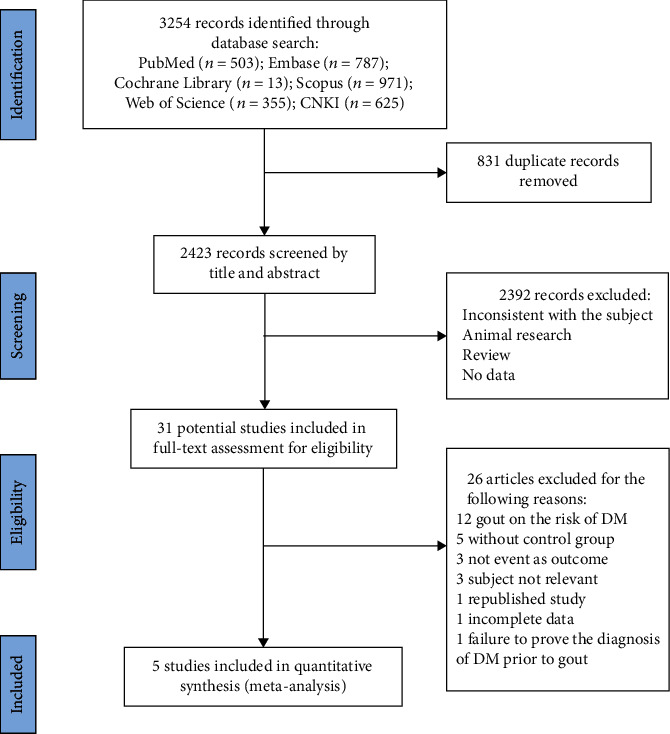
Flow chart of literature selection. CNKI: China National Knowledge Infrastructure; DM: diabetes mellitus.

By searching six databases, 3254 potentially relevant articles were identified (787 from Embase, 503 from PubMed, 13 from Cochrane Library, 971 from Scopus, 355 from Web of Science, and 625 from CNKI). After checking the records and removing duplicates, 2423 articles were screened by titles and abstracts. 2392 articles were removed due to irrelevant studies, leaving 31 articles for full-text review. 26 of the 31 articles were rejected for the following reasons: the effect of gout/hyperuricemia on the risk of DM (*n* = 12), not event as outcome (*n* = 3), subject not relevant (*n* = 3), studies without control group (*n* = 5), study failure to prove the diagnosis of DM prior to gout (*n* = 1), republished study (*n* = 1), and incomplete data (*n* = 1). Finally, five studies [11, 17, 18, 23, 24] met the inclusion criteria and were included in this meta-analysis. The screening process is shown in Figure 1.

### 3.2. Characteristics of the Included Studies

Three cohort studies [11, 18, 23] and two case-control studies [17, 24] involving 863,755 participants were included in this meta-analysis. The included studies were published from 2010 to 2016. Of the three cohort studies, two studies [11, 18] explored the relationship between DM and the risk of gout, while another study [23] showed the impact of T2DM on the risk of gout. Two case-control studies [17, 24] revealed the impact of T1DM and T2DM on the risk of gout, respectively. Four studies [11, 17, 18, 23] discussed the impact of gender differences of patients with DM on the risk of gout. All studies were based on large databases, and the diagnosis of DM or gout was mainly based on diagnostic codes, self-report, prescriptions for drug use, or laboratory findings. The quality of the included studies was evaluated according to NOS, and the NOS scores ranged from 6 to 8. The detailed characteristics of the included studies are illustrated in Tables 1 and 2.

### 3.3. Overall Meta-Analysis and Sensitivity Analysis

**Figure 2:**
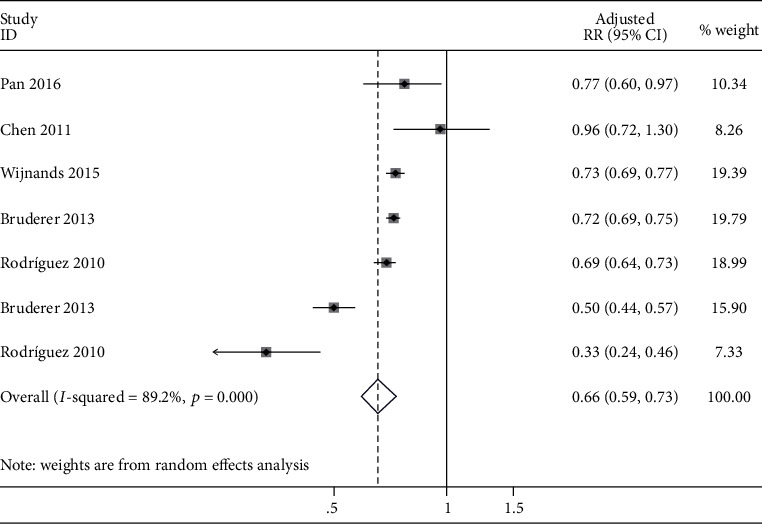
Forest plot of the risk of gout in patients with DM compared with controls. DM: diabetes mellitus; RR: relative risk; CI: confidence interval.

Given that two case-control studies [17, 24] discussed the impact of T1DM and T2DM on the risk of gout, respectively, therefore, each of the two studies combined the effect estimate according to two studies. Finally, a pooled effect estimate to assess the impact of DM on the risk of gout was calculated from seven studies with a total of 863,755 participants. Compared with the control groups, the pooled aRR of gout in patients with DM was 0.66 (95% CI: 0.59 to 0.73), but the heterogeneity was high (*I*^2^ = 89.2%), as shown in Figure 2. To identify sources of heterogeneity and assess the robustness of results, sensitivity analysis was performed by removing each study individually, and the estimated aRRs in the sensitivity analysis ranged from 0.64 (95% CI: 0.54 to 0.75) to 0.70 (95% CI: 0.64 to 0.77) (Table S1). Deletion of any single study did not change the overall statistical significance, showing that the results were steady and reliable in statistics. As less than 10 studies were included, no funnel plot was produced to assess publication bias.

### 3.4. Subgroup Analysis and Metaregression Analysis

To further explore sources of heterogeneity, a metaregression analysis and a subgroup analysis were performed according to geographical location, study design, and types of DM. In the subgroup analysis, the majority of strata showed inverse association between DM and the risk of gout. However, except for types of DM, there was no significant statistical significance between subgroups with a metaregression analysis, indicating that types of DM contributed the most to the heterogeneity (Table 3).

**Figure 3:**
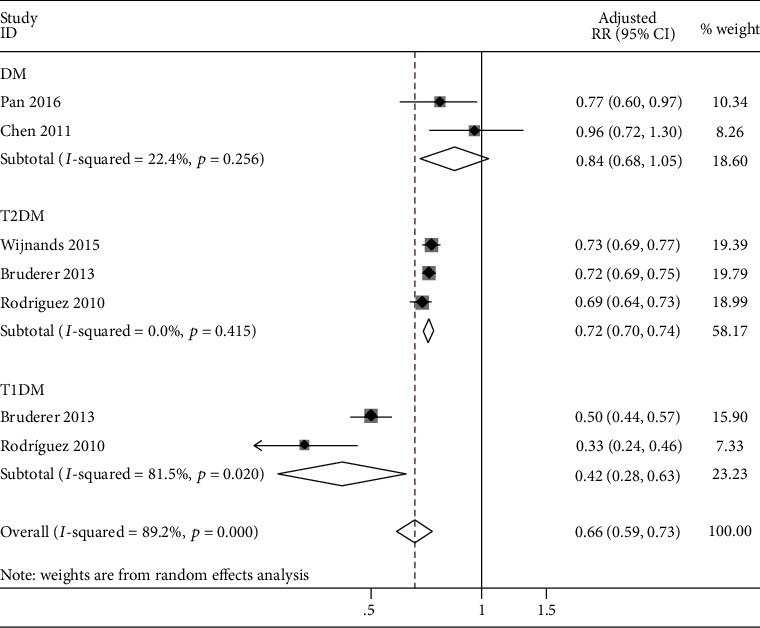
Subgroup analysis of the risk of gout in individuals with DM: grouped by types of DM. DM: diabetes mellitus; T2DM: type 2 diabetes mellitus; T1DM: type 1 diabetes mellitus; RR: relative risk; CI: confidence interval.

Subgroup analysis by types of DM (two studies in DM [11, 18], three studies in T2DM [17, 23, 24], and two studies in T1DM [17, 24]) showed that heterogeneity had a significant decrease in the DM subgroup (*I*^2^ = 22.4%) and the T2DM subgroup (*I*^2^ = 0%) but was still high in the T1DM subgroup (*I*^2^ = 81.5%). The risk of gout was significantly lower in T1DM than in T2DM; the pooled aRR in T1DM was 0.42 (95% CI: 0.28 to 0.63) and the aRR in T2DM was 0.72 (95% CI: 0.70 to 0.74), while no association was found in the DM subgroup (aRR: 0.84; 95% CI: 0.68 to 1.05), as shown in Figure 3.

### 3.5. Sex-Specific Analysis of DM and the Risk of Gout

**Figure 4:**
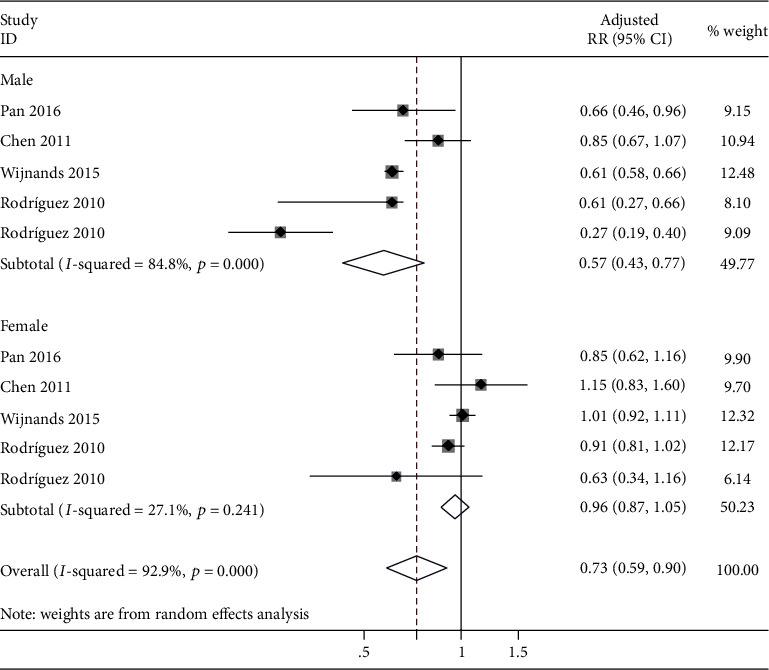
Sex-specific analysis of the risk of gout in individuals with DM. RR: relative risk; CI: confidence interval.

Considering the gender difference in the incidence of gout, further sex-stratified analysis was discussed between DM and the risk of gout. Four studies [11, 17, 18, 23] including 345,943 men and 334,752 women showed that there were inverse correlations between T2DM and the risk of gout among males; the pooled aRR was 0.57 (95% CI: 0.43 to 0.77), with high heterogeneity (*I*^2^ = 84.8%). However, the risk disappeared in females; the pooled aRR was 0.96 (95% CI: 0.87 to 1.05), with low heterogeneity (*I*^2^ = 27.1%) (Figure 4).

### 3.6. HbA1c Levels and the Risk Gout

**Figure 5:**
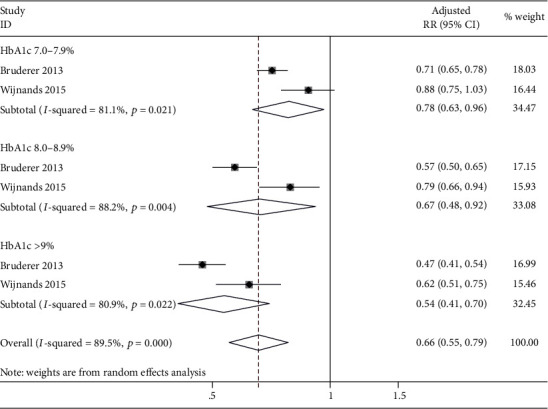
Forest plot of association between HbA1c level and the risk of gout. HbA1c: glycated hemoglobin; RR: relative risk; CI: confidence interval.

To evaluate the impact of HbA1c levels on the risk of gout, two studies [23, 24] were included and stratified according to HbA1c levels in DM. Interestingly, we found that HbA1c levels were inversely related to the risk of gout. Compared with HbA1c < 7.0%, the risk of gout was 22% reduced among those with HbA1c levels of 7.0-7.9% (aRR: 0.78; 95% CI: 0.63 to 0.96), 33% reduced among those with HbA1c levels of 8.0–8.9% (aRR: 0.67; 95% CI: 0.48 to 0.92), and even 46% reduced among those with HbA1c > 9% (aRR: 0.54; 95% CI: 0.41 to 0.70); however, there is high heterogeneity (Figure 5).

## 4. Discussion

The interplay between DM and gout is intricate. On the one hand, DM may be associated with an increased risk of gout, possibly due to DM-related comorbidities such as hypertension, obesity, and metabolic syndrome [3–7]. On the other hand, some pathophysiological mechanisms in DM may have the opposite effect on the risk of gout such as impaired inflammatory response and uricosuric effect of glycosuria [17, 25]. The results from this meta-analysis showed that patients with DM had a significantly lower risk of developing gout, especially in T1DM. However, contrary to our conclusions, four small cross-sectional studies suggested that DM was associated with an increased risk of gout [13–16], but since their results were not adjusted for the vital confounding factors of the coexistence of gout and DM, this could explain the positive associations. More importantly, three of these studies [13–15] did not have a control group and none of the studies [13–16] demonstrated that DM was diagnosed earlier than gout. Therefore, they did not meet our inclusion criteria and were not included in our meta-analysis. Moreover, a sex-specific association between DM and the risk of gout was found in this meta-analysis. The inverse association was observed in males only, not in females. In addition, increased HbA1c levels were associated with a reduced risk of gout in patients with DM.

However, high heterogeneity could not be ignored. Metaregression analysis revealed that types of DM were the source of heterogeneity. Although subgroup analysis by types of DM showed that the heterogeneity had a significant decrease in the DM subgroup (*I*^2^ = 22.4%) and the T2DM subgroup (*I*^2^ = 0%), there was still high heterogeneity in the T1DM subgroup (*I*^2^ = 81.5%) (Figure 3). So, the possible causes of high heterogeneity in the T1DM subgroup were further explored. This meta-analysis revealed that the inverse association was observed in males only (aRR: 0.57; 95% CI: 0.43 to 0.77) and not in females (aRR: 0.96; 95% CI: 0.87 to 1.05). Therefore, the higher male ratio with DM meant a lower risk of gout. Interestingly, the proportion of males (cases/controls—72.5%/73.9%) in the study by Rodriguez et al. [17] was significantly higher than that in other studies (Pan et al.: cases/controls—39.7%/39.8%; Wijnands et al.: cases/controls—49.4%/49.4%), as shown in Table 2, which might cause the lower risk of gout (aRR: 0.33; 95% CI: 0.24 to 0.46) and high heterogeneity in the T1DM subgroup. Unfortunately, the other study [24] in the T1DM subgroup did not provide the sex ratio of the participants, and even though we exerted every effort to contact the author, no sufficient raw data was obtained. More prospective cohort studies are needed to verify the results and provide more evidence. Another concern with subgroup analysis by types of DM was that the risk disappeared in the DM subgroup. It should be noted that of all the included studies, only the study by Chen et al. [18] found no negative correlation between DM and the risk of gout, finally leading to no statistical significance in the DM subgroup. So, the possible causes of this result were further analyzed. On the one hand, the choice of controls in this study [18] was not rigorous, which only took those without DM in the database as the control group; however, the important factors such as age and gender were not matched with the DM group. On the other hand, some important confounding factors were not adjusted, such as chronic kidney disease, chronic heart failure, and different types of diuretics, which were confirmed to be linked to a significant increase of the risk of gout in previous studies [26–28].

The reverse correlation between DM and the risk of gout may be explained by the uricosuric effect of glycosuria, which generally occurs when serum glucose levels exceed 10 mmol/L [25]. Some studies indicated that moderately elevated serum glucose levels were related to higher serum uric acid levels, while higher glucose levels sufficient to cause glycosuria (>10 mmol/L) were correlated with lower serum uric acid levels [25, 29, 30]. Indeed, it was observed that the excretion of uric acid was directly proportional to serum glucose levels once glucose load was enough to lead to glycosuria [25]. Moreover, people with prediabetes had a higher risk of developing gout, while the risk dropped to a lower level once they developed diabetes compared to nondiabetics [17, 31]. Similarly, several studies consistently have shown that people with DM have lower serum uric acid levels than normal individuals [29, 30, 32]. Therefore, glycosuria may increase the excretion of uric acid through a high filtration rate and osmotic diuresis, thus reducing serum uric acid level and the risk of gout in DM [25, 33].

Furthermore, an impaired inflammatory response may be another important reason for the lower risk of gout in DM. Gout is a common acute inflammatory arthritis caused by the deposition of monosodium urate crystals in joints. Urate crystals can rapidly trigger an inflammatory reaction by stimulating the synthesis and release of inflammatory mediators and then amplifying and maintaining a severe inflammatory response [34]. However, many inflammatory processes were found to be damaged in DM, which exactly hindered the intense inflammatory process caused by urate crystals [34]. The impaired inflammatory response found in DM included inhibition of leukocyte chemotaxis and increased leukocyte apoptosis [35, 36], decreased response of endothelial cells to permeability factors such as histamine and bradykinin [37], reduced mast cell degranulation [38], damaged adhesion of neutrophils to endothelial cells and migration to inflammatory sites [39, 40], and reduced release of cytokines and prostaglandin by neutrophils [41, 42]. These findings further provide a potential biological mechanism and may be the basis of the reverse association observed in this study.

In this meta-analysis, patients with high HbA1c levels or T1DM had a significantly reduced risk of gout, with possible mechanistic explanations such as a poorly controlled or long-lasting DM leading to a significantly uricosuric effect of glycosuria and a seriously impaired inflammatory response. As for the protective association observed only in males rather than females, the sex difference between serum uric acid levels and serum insulin levels may be a reasonable explanation. On the one hand, studies have shown that serum uric acid levels are independently and closely related to the degree of insulin resistance [43–45], and this correlation was significantly stronger among women than men [45]. Besides, since fasting serum insulin levels were significantly higher in both premenopausal and postmenopausal women with hyperuricemia than in men with hyperuricemia [45], sex hormones may also play a role. On the other hand, many studies have found that increased insulin concentration significantly promoted uric acid reabsorption via regulating urate transporter 1 (URAT1) and ATP-binding cassette subfamily G member 2 (ABCG2) in the kidney, thereby reducing urinary excretion of uric acid and increasing serum uric acid levels [43, 46–48]. Therefore, we hypothesize that the protective association in males is likely to be the residual association after cancelling out the effect of insulin on the reabsorption of uric acid. Whereas the effect of insulin on the reabsorption of uric acid was stronger in women than in men, we propose that the opposed effects just cancel each other out in females. Consistent with this gender difference, many evidences have confirmed that serum uric acid levels are predictive of coronary artery disease among women but not among men [49–51], and the sex differences between serum uric acid levels and serum insulin levels may be part of the reason for this difference in correlation [44]. However, it seems impossible to explain this gender difference of T1DM by the effect of insulin on the reabsorption of uric acid. Therefore, there may be other potential mechanisms for gender differences, which obviously need more studies to further confirm their existence.

Nevertheless, this study had some limitations. Firstly, all included studies were conducted using medical registration databases. The definitions of DM and gout were based on diagnostic codes or self-report, and it was inevitable to have some degree of misclassification. Such misclassification could also occur in the selection of the controls. For example, in the study by Wijnands et al. [23], the controls were individuals without a noninsulin antidiabetic drug or an insulin prescription during the whole study period, which might include a few individuals with DM.

Secondly, this was a meta-analysis of observational studies in which several potential confounding factors were adjusted to reduce confounding bias. However, due to some objective reasons of data collection, the confounding factors adjusted for each study were different and some unadjusted confounding factors in the original studies could not be ignored. Some comorbidities (such as hypertension, hyperlipidemia, congestive heart failure, and chronic kidney disease) [2, 28] and comedications (such as statins, low-dose aspirin, and especially different types of diuretics) [2, 27, 52] have been shown to be associated with a significantly increased risk of gout in previous studies. However, except for the study by Wijnands et al. [23] which adjusted both comorbidities and comedications as confounding factors, more comorbidities were adjusted but comedications were ignored in other studies. In addition, other important risk factors for gout, such as dietary exposure and physical activity, were not adjusted in most studies. Fortunately, all studies were adjusted by BMI associated with dietary habits and physical activity to control these risk factors to a certain extent. Moreover, the effects of antidiabetic drugs and uric acid-lowering drugs on the results also needed to be considered. Bruderer et al. [53] provided evidence that different types of antidiabetic drugs did not alter the risk of gout, while the use of urate-lowering drugs to treat hyperuricemia can affect the risk of gout in theory, and individuals with DM are generally more likely to treat hyperuricemia than nondiabetic individuals, as diabetic patients have more chance to discover hyperuricemia during the follow-up of the disease. Unfortunately, none of the included studies provided data on the use of uric acid-lowering drugs. Therefore, these results should be interpreted with caution, and more high-quality prospective cohort studies are needed in the future to provide more substantial evidence.

Thirdly, although we had tried our best to collect all available data, this meta-analysis included fewer original studies and had some degree of heterogeneity, even if we exerted our utmost effort to find the source of heterogeneity. Therefore, more prospective cohort studies are required in the future to confirm the results and more basic studies are needed to explore potential molecular mechanisms.

## 5. Conclusion

In conclusion, this meta-analysis suggested that DM reduced the future risk of gout and the protective effect was stronger in males, T1DM, or DM with a high HbA1c level. The substantial role of the uricosuric effect of glycosuria and the impaired inflammatory response might offer potential mechanisms. These findings may appear counterintuitive, but it is not contradictory to emphasize the prevention of DM and gout simultaneously. Gout should be prevented by dietary adjustments or treating hyperuricemia rather than by focusing on DM. Diabetes professionals should be aware of the relationship between DM and gout, especially in patients with well-controlled DM. These evidences might even change the treatment strategy of diabetic patients to use uric acid-lowering drugs more aggressively.

## Figures and Tables

**Table 1:** Characteristics of the studies included in the meta-analysis.

Author/year of publication	Study design	Data source	Study period	Definition of cases	Definition of controls	Ascertainment of DM/gout	Follow-up (cases / controls)
Pan [11] 2016	A prospective cohort study	China SCHS	1999-2010	Self-reported DM: face to face interviews to ask if they were told by their doctors that they had diabetes. If the response was “yes,” the participants would be included and be asked about the age of first diagnosis.	Participants who were randomly selected from the same database, reported to be free of DM, and were below the HbA1c cut off.	The diagnosis of gout was based on joint pain and swelling attributed to reported hyperuricemia by their physicians.	6.9/6.9
Chen [18] 2011	A prospective cohort study	Taiwan NHI	1994-2002	DM was defined as fasting blood sugar ≥126 mg/dL or use of antidiabetic medications.	Participants who were randomly selected from the same database and reported to be free of DM.	Gout is diagnosed by using the ICD-9 code.	7.31/7.31
Wijnands [23] 2015	A retrospective cohort study	UK CPRD GOLD	2004-2012	T2DM: received at least 1 prescription for a noninsulin antidiabetic drug (NIAD) recorded.	Sex, year of birth, and practice of history in the database-matched subjects without an NIAD or insulin prescription during the whole study period, who were randomly selected from the same database.	Gout is diagnosed by using READ codes.	4.3/4.5
Rodríguez [17] 2010	A case-control study	UK THIN	2000-2007	Gout is diagnosed by using READ codes.	Controls were frequency-matched to cases by age within one year, sex, and calendar year and were randomly selected from the same database.	Diagnostic code of the database. The type of diabetes is defined by the recorded code or age or medication.	Not applicable
Bruderer [24] 2013	A case-control study	UK GPRD	1995-2009	All patients aged between 18 and 80 years with an incident diagnosis of gout.	Age, sex, general practice, calendar time, and years of history in the database-matched subjects without a diagnosis of gout, who were randomly selected from the same database.	T1DM: diabetic patients with insulin use only; T2DM: diabetic patients treated with diet only and using oral antidiabetic drugs with or without concomitant use of insulin.	Not applicable

Abbreviations: SCHS—Singapore Chinese Health Study; UK—United Kingdom; CPRD GOLD—the UK Clinical Practice Research Datalink GOLD; Taiwan NHI—Taiwan's National Health Insurance; GPRD database—the UK-based General Practice Research Database; THIN database—the Health Improvement Network database; ICD-9 code—ninth version of the International Classification of Disease code; DM—diabetes mellitus; T1DM—type 1 diabetes; T2DM—type 2 diabetes.

**Table 2:** Characteristics of the studies included in the meta-analysis and quality assessment.

Author/year of publication	Cases (*n*)	Controls (*n*)	Male (%): cases/controls	Age: (mean ± SD): cases/controls	Type of DM	Adjustment by	Adjusted OR/RR/HR (95% CI)	NOS		
								Selection	Comparability	Outcome
Pan [11] 2016	3849	27288	39.7/39.8	62.1 ± 7.2/60.3 ± 7.3	DM	Age, sex, dialect, year of interview, educational level, moderate physical activity, strenuous sports, vigorous work, smoking status, alcohol use, body mass index, and history of hypertension	DM, RR 0.77 (0.60-0.97) Male 0.66 (0.46-0.96) Female 0.85 (0.62-1.16)	3	2	3
Chen [18] 2011	132556		NA	NA	DM	Age, sex, obesity, hypertension, hyperlipidemia, alcohol drinking, and cigarette smoking	DM, HR 0.96 (0.72-1.30) Male 0.85 (0.67-1.07) Female 1.15 (0.83-1.60)	3	1	3
Wijnands [23] 2015	221117	221117	49.4/49.4	60.4 ± 15.4/60.4 ± 15.4	T2DM	Age, sex, smoking status, alcohol use, postmenopausal status/oophorectomy, BMI, eGFR, hypertension, renal transplantation, diuretics, statins, low-dose aspirin, cyclosporine, and tacrolimus	T2DM, HR 0.73 (0.69-0.77) Male 0.61 (0.58-0.66) Female 1.01 (0.92-1.11)	3	2	3
Rodríguez [17] 2010	24768	50000	72.5/73.9	NA	T1DM T2DM	Sex, age, calendar year, GP visits, BMI, alcohol consumption, smoking, IHD, hypertension, hyperlipidemia, and renal failure	T1DM, OR 0.33 (0.24-0.46) Male 0.27 (0.19-0.40) Female 0.63 (0.34-1.16) T2DM, OR 0.69 (0.64-0.73) Male 0.61 (0.27-0.66) Female 0.91 (0.81-1.02)	2	2	3
Bruderer [24] 2013	91530	91530	NA	NA	T1DM T2DM	BMI, smoking, alcohol consumption, ischemic heart disease, congestive heart failure, hypertension, and chronic kidney disease	T1DM, OR 0.50 (0.44-0.57) T2DM, OR 0.72 (0.69-0.75)	3	2	1

Abbreviations: NOS—Newcastle-Ottawa Quality Assessment Scale; BMI—body mass index; eGFR—estimated glomerular filtration rate; GP—general practitioner; IHD—ischaemic heart disease; DM—diabetes mellitus; T1DM—type 1 diabetes; T2DM—type 2 diabetes; OR—odds ratio; RR—relative risk; HR—hazard ratio; CI—confidence interval; NA—not available.

**Table 3:** Stratified meta-analysis and metaregression of the association of DM and the risk of gout.

Covariates	No. of study	RR (95% CI)	*I* ^2^ (%)	Ph^∗^	Metaregression		
					Tau^2^	Adj − *R*^2^ (%)	Ph^∗∗^
Overall	7	0.66 (0.59, 0.73)	89.2	<0.000			
Subgroup analyses							
Types of DM					0 .000	99.6	0.01
DM	2	0.84 (0.68, 1.05)	22.4	0.256			
T1DM	2	0.42 (0.28, 0.63)	81.5	0.020			
T2DM	3	0.72 (0.70, 0.74)	0.0	0.415			
Study design					0.01	26.2	0.149
Cohort study	3	0.77 (0.68, 0.88)	39.7	0.191			
Case-control study	4	0.58 (0.48, 0.69)	93.7	<0.000			
Geographical location					0.072	16.9	0.208
Asia	2	0.84 (0.68, 1.05)	22.4	0.256			
Europe	5	0.62 (0.55, 0.70)	92.1	<0.000			

Abbreviations: RR—relative risk; CI—confidence interval; Ph^∗^—*p* value for heterogeneity within each subgroup; Ph^∗∗^—*p* value for heterogeneity between subgroups in metaregression analysis.

## Supplementary Materials

Supplementary 1 Table S1. Sensitivity analysis of the risk of gout in DM patients and controls.

Supplementary 2 Excel S1-S2. The retrieval history in PubMed and EMBASE databases.
