# Supplementary material for: Diabetes Mellitus Is Associated with a Lower Risk of Gout: A Meta-Analysis of Observational Studies
Source: J Diabetes Res. 2020 Jul 9;2020:5470739. doi: 10.1155/2020/5470739 (PMC7369651; doi:10.1155/2020/5470739)
Supplement: Supplementary 1 — Table S1. Sensitivity analysis of the risk of gout in DM patients and controls. [file 5470739.f1.doc]

**Table S1.** Sensitivity analysis of the risk of gout in DM patients and healthy controls

| [S](../../../../C:/Program%20Files%20(x86)/Youdao/Dict/8.8.0.0/resultui/html/index.html#/javascript:;)ensitivity [A](../../../../C:/Program%20Files%20(x86)/Youdao/Dict/8.8.0.0/resultui/html/index.html#/javascript:;)nalysis | RR (95%CI) | I^2^ (%) | Ph |
| --- | --- | --- | --- |
| All | 0.66 (0.59, 0.73) | 89.2 | <0.000 |
| removing An Pan 2016 | 0.65 (0.57, 0.73) | 90.9 | <0.000 |
| removing Wijnands 2015 | 0.64 (0.55, 0.74) | 90.6 | <0.000 |
| removing Jiunn-Horng Chen 2011 | 0.64 (0.57, 0.71) | 90.2 | <0.000 |
| removing Saskia G Brudere 2013 | 0.64 (0.54, 0.75) | 90.6 | <0.000 |
| removing Luis A 2010 | 0.65 (0.56, 0.75) | 90.9 | <0.000 |
| removing Luis A 2010 | 0.69 (0.63, 0.76) | 85.6 | <0.000 |
| removing Saskia G Bruderer 2013 | 0.70 (0.64, 0.77) | 81.9 | <0.000 |

RR, relative risk; CI, confidence interval; Ph, p-value for heterogeneity within each group.
